# No association of *GSTP1* rs1695 polymorphism with amyotrophic lateral sclerosis: A case-control study in the Brazilian population

**DOI:** 10.1371/journal.pone.0247024

**Published:** 2021-02-19

**Authors:** Jéssica Barletto de Sousa Barros, Kamilla de Faria Santos, Rômulo Morais Azevedo, Rayana Pereira Dantas de Oliveira, Ana Carolina Dourado Leobas, Dhiogo da Cruz Pereira Bento, Rodrigo da Silva Santos, Angela Adamski da Silva Reis

**Affiliations:** 1 Laboratory of Molecular Pathology, Department of Biochemistry and Molecular Biology, Institute of Biological Sciences, Federal University of Goiás (UFG), Goiânia, Goiás, Brazil; 2 Rehabilitation and Readaptation Medical Center Dr. Henrique Santillo (CRER), Goiânia, Goiás, Brazil; First Affiliated Hospital of Dalian Medical University, CHINA

## Abstract

Amyotrophic Lateral Sclerosis (ALS) is a rare neurodegenerative disease that affects motor neurons and promotes progressive muscle atrophy. It has a multifactorial etiology, where environmental conditions playing a remarkable role through the increase of oxidative stress. Genetic polymorphisms in cell detoxification genes, such as *Glutathione S-Transferase Pi 1* (*GSTP1*) can contribute to excessive oxidative stress, and therefore may be a risk factor to ALS. Thus, this study aimed to investigate the role of the *GSTP1* rs1695 polymorphism in ALS susceptibility in different genetic inheritance models and evaluate the association of the genotypes with risk factors, clinical and demographic characteristics of ALS patients from the Brazilian central population. This case-control study was conducted with 101 patients with ALS and 101 healthy controls. *GSTP1* rs1695 polymorphism genotyping was performed with Polymerase Chain Reaction–Restriction Fragment Length Polymorphism (PCR-RFLP). The statistical analysis was carried out using the SPSS statistical package and SNPStats software. Analysis of genetic inheritance models was performed by logistic regression, which was used to determine the Odds Ratio. The results of this first study in the Brazilian population demonstrated that there was no risk association between the development of ALS and the *GSTP1* rs1695 polymorphism in any genetic inheritance model (codominant, dominant, recessive, overdominant, and logarithmic); and that the polymorphic variants were not associated with the clinical and demographic characteristics of ALS patients. No association of the *GSTP1* rs1695 polymorphism and ALS development in the Brazilian central population was found. These findings may be justified by the multifactorial character of the disease.

## Introduction

Amyotrophic Lateral Sclerosis (ALS) is a devastating neurodegenerative disorder that affects motor neurons in the motor cortex, brainstem, and spinal cord [1]. Typically, the disease course is rapid, within 3–5 years of symptom onset, and involves signs and symptoms of progressive muscle atrophy, weakness, paralysis, and respiratory failure, which results in the individual’s death [2, 3].

ALS worldwide incidence ranges from 1 to 2.6 per 100,000 individuals and in Brazil according to a study conducted in São Paulo city, the incidence and prevalence rates were estimated in 0.4 cases/100,000 persons/year and 0.9–1.5 cases/100,000 persons, respectively [4, 5]. Despite has been considered rare, studies suggest that the worldwide incidence will increase by approximately 70% in the next two decades and future projections estimate that 400,000 diagnoses will be carried out worldwide in 2040 [6, 7].

In addition, due to the lack of cure and the considerable personal, societal, and economic impact, studies have been developed to elucidate the etiology and pathogenic mechanisms of the disease [8–10]. Until now, it is clearly known that etiologically the vast majority of cases are sporadic (90–95%), in which there is an interaction between genetic and environmental conditions that promote the development of the disease in genetically predisposed individuals [4, 11]. Exposure environmental had been hypothesized as an important role in the pathogenesis of ALS associated mainly with the increase of oxidative stress [9, 11, 12].

*Glutathione S-Transferases (GSTs)* genes are responsible for coding enzymes involved in the detoxification of toxic products, protecting the organism against oxidative damage [12, 13]. The members of the GST family have genetic polymorphisms, wich promote a complete lack or reduction of enzyme function. Consequently, carriers of lacking GST isoenzyme activity have been altered the capacity for protecting cellular from oxidants agents [14].

Glutathione S-Transferase Pi 1 (GSTP1) isoenzyme is most often produced in the brain where it might alter the effectiveness of neurotoxins and products of oxidative stress contributing to detoxification and cell protection [15]. Hence, the presence of *GSTP1* rs1695 polymorphism may contribute to the development of neurodegenerative diseases through excessive oxidative stress associated with pathogenic mechanisms that could promote the death of the motor neurons [12, 13, 15, 16].

*GSTP1* rs1695 variant (chromosome 11q13; gene identity 2950, MIM 134660; https://www.ncbi.nlm.nih.gov/gene/2950) [16] consists of an adenine to guanine transition at position 313 (codon 105), which promotes substitution of amino acid isoleucine (Ile) for valine (Val), resulting in a lower activity of the Pi isoenzyme. Thus, carriers of the G/G genotype have a lower Pi isoenzyme activity than carriers of the A/A genotype, while heterozygotes individuals (A/G) have an intermediate activity [15, 17].

The role of the *GSTP1* rs1695 polymorphism has already been investigated in different neurodegenerative disorders, such as Alzheimer, Parkinson, and Multiple Sclerosis [13, 15, 18]. Although the role of GST polymorphism in ALS is scarce in the literature, none has linked *GSTP1* rs1695 polymorphism in the different models of genetic inheritance. Furthermore, until the moment there is no report regarding the association of *GSTP1* rs1695 in the Brazilian population, especially from the Central-Western region.

Additionally, this first study in the Brazilian central population was designed to investigate the role of the *GSTP1* rs1695 polymorphism in ALS susceptibility in different genetic inheritance models, as well as associate the polymorphic variants to risk factors, demographic and clinical characteristics. This has prompted us to assess a possible association between correlate with a biomarker of oxidative stress in patients with ALS. Our findings can help the understanding of the role of the *GSTP1* rs1695 polymorphism in ALS pathogenesis.

## Materials and methods

### Statement of ethics

This study was conducted according to the Research Ethics Committee (number CAAE 79593117.7.0000.5083) of the Federal University of Goiás and in accordance with the Ethical Principles for Medical Research Involving Human Beings of the Declaration of the World Medical Association of Helsinki. All participants provided written informed consent. If necessary, we send the approbation letter. Requests for data should be sent to the following address: Research Ethics Committee—Federal University of Goiás, Goiânia, Goiás, Brazil (Zipcode: 74690–631), Tel: 55 (62) 3521–1215 /55 (62) 3521–2045. E-mail: cep.prpi@ufg.br

### Study subjects

The study included 101 patients with ALS confirmed for the diagnosis at the Rehabilitation and Readaptation Medical Center Dr. Henrique Santillo (CRER), Goiânia—GO, Brazil, and 101 healthy controls recruited at the Clinics Hospital from the Faculty of Medicine—Federal University of Goiás, Goiânia—GO, Brazil.

Among the inclusion criteria for the case group were minimum and maximum age of 18 and 90 years, respectively; and ALS diagnosis (regardless of the stage of the disease) according to the laboratory and imaging guidelines established by CRER. Incomplete CRER protocol, diagnosis of other neurodegenerative diseases, and impossibility of blood collection were considered exclusion criteria.

All individuals of the study answered a questionnaire approaching aspects such as age, gender, smoking, and alcohol intake. Among the individuals of the control group, we considered smokers and alcohol consumers those who used the substances at least one year before the study. Patients with ALS who reported consuming cigarettes and alcohol at least one year before the diagnosis were considered smokers and alcohol consumers.

ALS patients still answered questions related to risk factors, such as physical activity and occupation. Medical records were also consulted for analysis of peculiar characteristics of the disease, such as diagnostic time, data and initial time of symptoms, use of riluzole, presence of comorbidities, use of mechanical ventilation, and others.

Furthermore, to present a better result of genetic association both groups were matched by gender and age, eliminating the possibility of a confounding factor. The inclusion criteria were established according to The STrengthening the REporting of Genetic Association studies (STREGA) guidelines for improved reporting of genetic association studies [19].

### Sample collection and *GSTP1* rs1695 polymorphism analysis

Peripheral venous blood was collected into ethylenediaminetetraacetic acid-coated tubes. After collection, the samples were centrifuged at 5000 rpm for 10 minutes and then subjected to DNA extraction with the commercial kit (Invitrogen®), according to the manufacturer’s instructions. The extracted DNA was stored at -80°C and subsequently subjected to quantification by the NanoDrop™ ND-1000 spectrophotometer (ThermoFisher®, USA).

*GSTP1* rs1695 polymorphism genotyping was performed through Polymerase Chain Reaction–Restriction Fragment Length Polymorphism (PCR-RFLP) using previously described primers by Harries et al [20].

PCR product (176 base pairs) was digested with 1.0 units of Alw26I enzyme (Thermo Scientific TM®) and run on 12% polyacrylamide gel electrophoresis followed by the silver nitrate staining. The presence of three fragments (85, 91, and 176 base pairs) indicated the genotype as heterozygous (A/G), while that two fragments (85 and 91 base pairs) revealed mutant genotype (G/G). The wild genotype (A/A) had been shown by the presence of only one fragment (176 base pairs), as shown in S1 Fig. The details of the protocol are available at protocols.io (dx.doi.org/10.17504/protocols.io.bqgqmtvw). All genetic analyzes were performed at the Laboratory of Molecular Pathology, Institute of Biological Sciences (ICB), Federal University of Goiás, Goiânia, GO, Brazil.

### Statistical analysis

Statistical analysis was performed using the SPSS statistical package (version 23, Chicago, Illinois, USA) and the SNPStats software (available at https://www.snpstats.net/start.htm). The data normality was verified by the Kolmogorov-Smirnov test and the general characteristics of the study population were compared by Pearson’s chi-squared (χ2) and when necessary, the Student t-test was applicable.

Frequencies of the *GSTP1* rs1695 polymorphism were estimated using the Hardy Weinberg equilibrium (HWE) and the comparison of the observed and expected genetic frequencies were calculated by exact test. The allelic association was evaluated by Fisher’s exact test, and analyzes of genetic inheritance models (codominant, dominant, recessive, overdominant, and logarithmic) were performed by logistic regression, which was used to determine the Odds Ratio (OR). Akaike Information Criteria (AIC) and the Bayesian Information Criteria (BIC) were also applied to select the best genetic model of inheritance.

The genotypes of the *GSTP1* rs1695 polymorphism were associated with demographic and clinical variables of the ALS patients by the Post Hoc chi-square test, being all p-values corrected by the Bonferroni adjustment for multiple comparisons tests. Age was also evaluated through analysis of variance (ANOVA), and the Kaplan-Meier curves was applied to assess cumulative survival according to age at diagnosis, time from symptom to diagnosis, symptom onset to outcome, and diagnosis to outcome. All these variables were evaluated by the genotypes. In all results, a significance level of 5% was used.

## Results

The 101 patients with ALS and the 101 healthy controls were characterized according to age, gender, alcohol intake, and smoking, as shown in Table 1. The mean age was 57.3 for ALS patients and 57.1 for the control group. We observed a male predominance in the case group (55.4%). Besides, alcohol consumption (46.0%) was higher than smoking (36.0%) among ALS patients. None of these variables showed a significant association with the disease (p>0.05).

**Table 1:** General characteristics of the study population.

	n (%)		Total 202 (100)	p
	Control 101 (50)	ALS 101 (50)		
**Age (Mean ± SD)**	57.1 ± 10.8	57.3 ± 12.9	57.2 ± 11.9	0.362^a^
**Gender**				
Female	45/101 (44.6%)	45/101 (44.6%)	90/202 (44.6%)	1.000^b^
Male	56/101 (55.4%)	56/101 (55.4%)	112/202 (55.4%)	
**Alcohol Intake**				
No	53/85 (62.4%)	54/100 (54.0%)	107/185 (57.8%)	0.319 ^b^
Yes	32/85 (37.6%)	46/100 (46.0%)	78/185 (42.2%)	
**Smoking**				
No	48/85 (56.5%)	64/100 (64.0%)	112/185 (60.5%)	0.372 ^b^
Yes	37/85 (43.5%)	36/100 (36.0%)	73/185 (39.5%)	

^a^Student t-test; ^b^Pearson’s chi-squared. Abbreviation: SD = Standard Deviation. Significance between groups: p*<*0.05.

Regarding the distribution, the genotypes frequencies in the case group were 52.5% (A/A), 41.6% (A/G) and 5.9% (G/G); while in the control group the frequencies were 41.5% (A/A), 46.5% (A/G) and 12% (G/G). Moreover, it was found that 27% and 35% of the case and control group had the mutant allele (G), respectively (Table 2).

**Table 2:** Genotypic and allele frequency of the *GSTP1* rs1695 polymorphism in ALS patients and controls.

*GSTP1* rs1695	n (%)	
	Control 101	ALS 101
**Genotype frequency**		
A/A	42 (41.5)	53 (52.5)
A/G	47 (46.5)	42 (41.6)
G/G	12 (12.0)	6 (5.9)
**Allele frequency**		
A	131 (65.0)	148 (73.0)
G	71 (35.0)	54 (27.0)

The observed and expected genotypic frequencies of the case, control group, and the total study population were also compared and are described in Table 3. These findings showed that these were within the parameters of the Hardy-Weinberg equilibrium (HWE) (p>0.05).

**Table 3:** Genotypic frequencies: Observed and expected for the case, control group, and the total study population, and the Hardy-Weinberg equilibrium test.

Group (n)	Genotype	Observed	Expected	p-value^a^
**Control (101)**	A/A	42	42.5	1.000
	A/G	47	46.0	
	G/G	12	12.5	
**Case (101)**	A/A	53	54.2	0.62
	A/G	42	39.6	
	G/G	6	7.2	
**Total (202)**	A/A	95	96.3	0.74
	A/G	89	86.3	
	G/G	18	19.3	

^a^ Exact Test for Hardy-Weinberg equilibrium. Significance between groups: p*<*0.05.

Furthermore, to verify the association of the *GSTP1* rs1695 polymorphism with susceptibility to ALS, an analysis of genetic inheritance models was performed (Table 4). The results showed no significant difference between the case and control group for all models (codominant, dominant, recessive, overdominant, and logarithmic), p> 0.05. No risk association was found between the *GSTP1* rs1695 polymorphism and ALS. The same was found for the analysis of allelic association, the mutant allele (G) did not present risk for ALS susceptibility (p = 0.085), Table 4.

**Table 4:** Comparison of the genetic inheritance models for *GSTP1* rs1695 polymorphism and allelic association in ALS patients.

Models	Genotype	Control (101)	Case (101)	OR (CI 95%)	p^a^	AIC	BIC
**Codominant**	A/A	42 (41.6%)	53 (52.5%)	1.00	0.170	282.4	292.4
	A/G	47 (46.5%)	42 (41.6%)	0.71 (0.40–1.27)			
	G/G	12 (11.9%)	6 (5.9%)	0.40 (0.14–1.14)			
**Dominant**	A/A	42 (41.6%)	53 (52.5%)	1.00	0.120	281.6	288.2
	A/G-G/G	59 (58.4%)	48 (47.5%)	0.64 (0.37–1.12)			
**Recessive**	A/A-A/G	89 (88.1%)	95 (94.1%)	1.00	0.140	281.8	288.4
	G/G	12 (11.9%)	6 (5.9%)	0.47 (0.17–1.30)			
**Overdominant**	A/A-G/G	54 (53.5%)	59 (58.4%)	1.00	0.480	283.5	290.1
	A/G	47 (46.5%)	42 (41.6%)	0.82 (0.47–1.43)			
**Logarithmic**				0.66 (0.43–1.03)	0.062	280.6	287.2
**Allele**							
	A	131 (65%)	148 (73%)		0.085		
	G	71 (35%)	54 (27%)				

^a^The comparison of the genetic inheritance models was obtained by logistic regression and the allelic association was evaluated by Fisher’s exact test. All study participants were matched by gender and age, and therefore this adjustment was not necessary for logistic regression. Abbreviations: OR = Odds Ratio; CI = Confidence Interval 95%; AIC = Akaike Information Criteria; BIC = Bayesian Information Criteria. Significance between groups: p*<*0.05.

**Fig 1:**
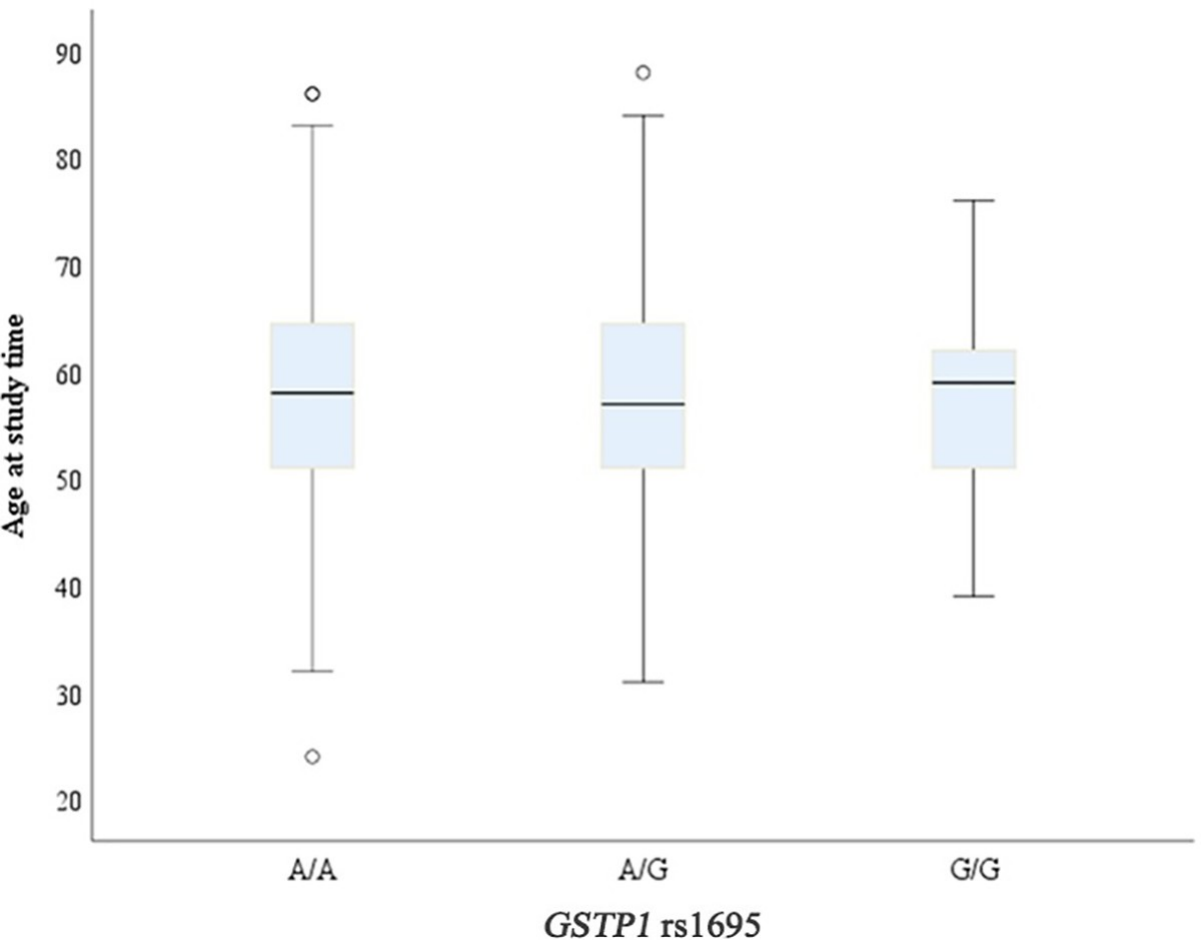
Boxplot graph comparing age with *GSTP1* rs1695 polymorphism genotypes in ALS patients.

An age comparison was performed for ALS patients and *GSTP1* rs1695 genotypes. According to the results carriers of the different genotypes (A/A, A/G, and G/G) had similar mean age, and therefore no statistical difference was found (p = 0.69) (Table 5, Fig 1).

**Table 5:** Comparison of age with different genotypes of the *GSTP1* rs1695 polymorphism in ALS patients.

*GSTP1* rs1695	Age (years)		p^a^
	Average	Standard Deviation	
**A/A**	57.47	1.18	0.69
**A/G**	57.69	11.36	
**G/G**	58.81	9.44	

^a^ANOVA. Significance between groups: p*<*0.05.

In addition, the genotypes of the *GSTP1* rs1695 polymorphism were associated with the outcome in ALS patients, which could be positive (survival) and negative (death). The results revealed that most of the individuals who manifested a positive outcome had A/A genotype (54.4%) and that a higher percentage of patients with a negative outcome (54.5%) had A/G genotype. However, no significant difference was found.

Furthermore, we verified if mutant allele (G) demonstrated a relationship with death in ALS patients. The results showed that the wild allele (A) was higher for the positive outcome (74,4%), although without no significant association (p = 0.28), demonstrating, therefore, that the mutant allele (G) was not associated with death causality. These results are described in Table 6.

**Table 6:** Association between *GSTP1* rs1695 polymorphism genotypes with the outcome in ALS patients.

*GSTP1* rs1695	Outcome n (%)		Total	p
	Negative	Positive		
**Genotypes**				
A/A	4 (36.4)	49 (54.4)	53 (52.5)	0.27^a^
A/G	6 (54.5)	36 (40.0)	42 (41.6)	0.37^a^
G/G	1 (9.1)	5 (5.6)	6 (5.9)	0.62^a^
**Alleles**				
A	14 (63.6)	134 (74.4)	148 (73.3)	0.28^b^
G	8 (36.4)	46 (25.6)	54 (26.7)	

^a^Post Hoc chi-square test (p-values were corrected by the Bonferroni adjustment for multiple comparisons tests)

^b^ Pearson’s chi-squared. Abbreviation: n = Absolute Frequency. Significance between groups: p*<*0.05

Risk factors and the demographic and clinical profile of ALS patients were also associated with different *GSTP1* rs1695 genotypes (Table 7). Variables such as gender, ethnicity, physical activity, alcohol intake, smoking, disease classification, family history, riluzole use, complications, and previous pathologies were not associated with any genotype. However, environmental exposure was more prevalent in individuals with A/A genotype (39.6%) than in A/G (16.7%) and G/G (16.7%), demonstrating that there is a significant difference between the A/A genotype of the *GSTP1* rs1695 polymorphism with environmental exposure (p = 0.03).

**Table 7:** Association between *GSTP1* rs1695 genotypes and the characteristics of the ALS patients.

Variables	*GSTP1* n (%)			p^a^
	A/A	A/G	G/G	
**Gender**				
Female	24 (45.3)	18 (42.9)	3 (50.0)	0.93
Male	29 (54.7)	24 (57.1)	3 (50.0)	
**Physical Activity**				
No	24 (45.3)	21 (50.0)	2 (33.3)	0.72
Yes	29 (54.7)	21 (50.0)	4 (66.7)	
**Environmental exposure**				
No	32 (60.4)	35 (83.3)	5 (83.3)	0.03*
			
Yes	21 (39.6)	7 (16.7)	1 (16.7)	
**Alcohol Intake**				
No	29 (54.7)	22 (52.4)	4 (66.7)	0.80
Yes	24 (45.3)	20 (47.6)	2 (33.3)	
**Smoking**				
No	34 (64.2)	29 (69.0)	2 (33.3)	0.23
Yes	19 (35.8)	13 (31.0)	4 (66.7)	
**Classification**				
Sporadic ALS	52 (98.1)	37 (88.1)	6 (100.0)	0.10
Familiar ALS	1 (1.9)	5 (11.9)	0 (0.0)	
**Neurological Disease in Family**				
No	30 (56.6)	26 (61.9)	4 (66.7)	0.81
Yes	23 (43.4)	16 (38.1)	2 (33.3)	
**Complications**				
No	38 (71.7)	29 (69.0)	4 (66.7)	0.94
Yes	15 (28.3)	13 (31.0)	2 (33.3)	
**Previous pathologies**				
No	28 (52.8)	25 (59.5)	1 (16.7)	0.14
Yes	25 (47.2)	17 (40.5)	5 (83.3)	
**Riluzole Use**				
No	11 (20.8)	11 (26.2)	3 (50.0)	0.27
Yes	42 (79.2)	31 (73.8)	3 (50.0)	

*Significant difference between groups (p*<*0.05). ^a^Post Hoc chi-square test. All p-values were corrected by the Bonferroni adjustment for multiple comparisons tests. Abbreviation: n = Absolute Frequency. Significance between groups: p*<*0.05.

**Fig 2:**
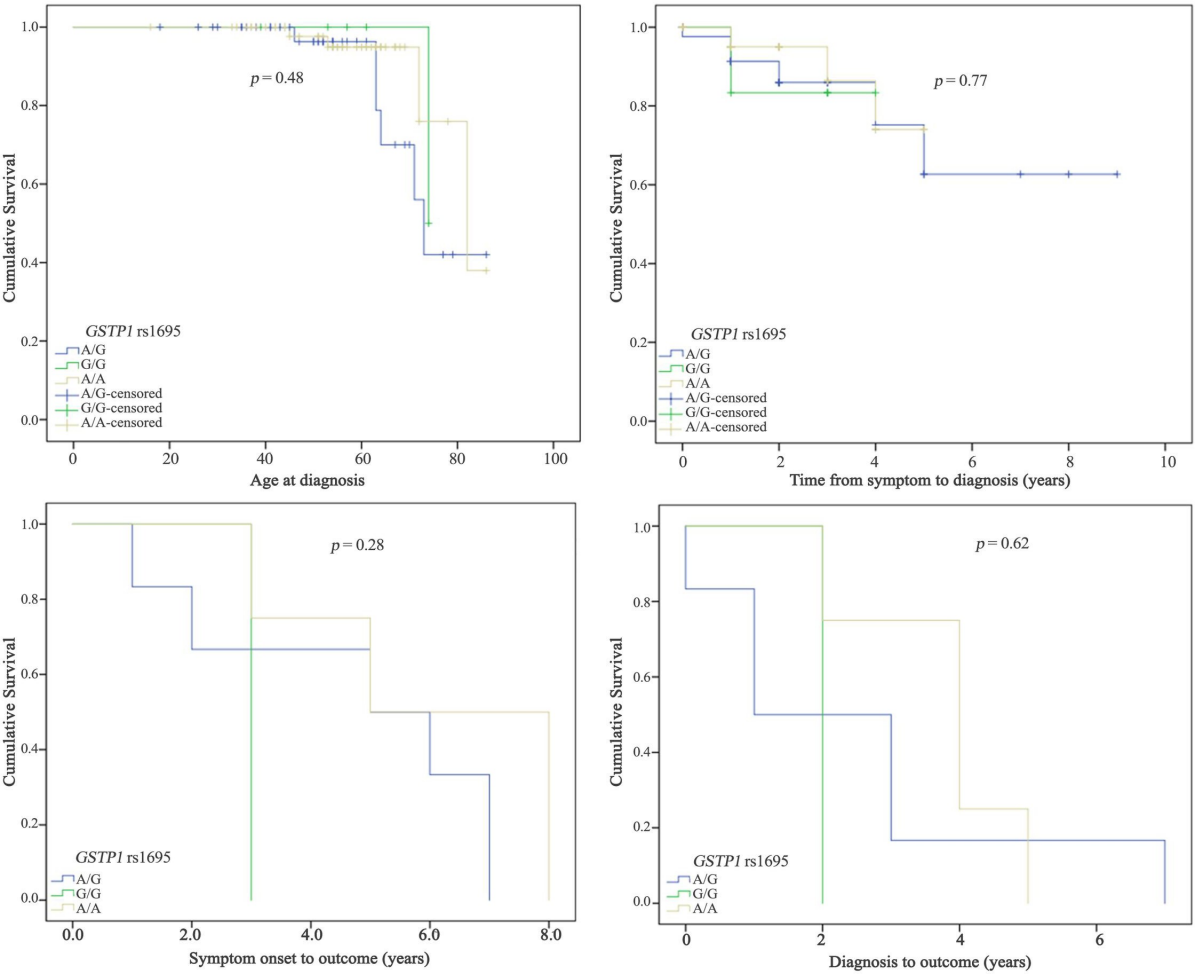
Kaplan-Meier curves to determine the clinical characteristics of ALS patients according to genotypes.

The Kaplan-Meier curves was also used to assess cumulative survival according to age at diagnosis, time from symptom to diagnosis, symptom onset to outcome, and diagnosis to outcome. This analysis did not demonstrate significant differences, p-value >0.05, Fig 2.

## Discussion

ALS is a progressive neurodegenerative disease that promotes a considerable social and economic impact [3]. Although the disease is rare, it has been increasing in recent years due to the aging of the population, since this is one of the main aspects of risk involved in the neurodegenerative context [6].

In addition, studies show that ALS is more prevalent between 50 and 75 years [6, 11]; and a Brazilian study conducted in Paraná and Rio de Janeiro showed an average age of 54.4 years and 53.6 years for ALS patients, respectively; corroborating with our findings (Table 1) [21, 22].

A higher frequency of the disease in men (55,4%) was also evidenced (Table 1). Men prevalence in ALS is commonly reported in the literature [9, 11] and was also demonstrated a male / female ratio of 1.6: 1 in a study carried out in Minas Gerais (Brazil) [4]. Despite this, the mechanisms behind gender and ALS are not clearly elucidated. However, some studies has been justified these differences by hormonal conditions between gender [23, 24].

Thus, growing evidences suggest that androgens have a toxic potential to the organism, while estrogens act as protective factors to ALS [25, 26]. Estrogen stands out for its potent neuroprotective action, related to neuronal differentiation and plasticity, as well as by antioxidant actions that remove reactive oxygen species and inhibits the synthesis of inflammatory cytokines, such as TNF-α, IL-1β and IL-6, which would contribute to neurological preservation in women [24].

On the other hand, although studies show a higher prevalence of ALS in Caucasians, our study did not analyze the *GSTP1* rs1695 polymorphism in different ethnicities of the Brazilian population due to the intense admixture. The Brazilian population has a great genetic diversity as a result of the three main ancestral contributions (Native Americans, Europeans, and Africans), and the high miscegenation [27, 28]. Moreover, Brazil is a wide territory, where different regions had different colonizers, which reflects in a genetic background variance of the population [27, 29]. This results in a large variability of skin pigmentation, which promotes a categorization of ethnicity not based on ancestry, but on the physical features of the individual once in Brazil ethnic stratification is phenotypically characterized by skin color [27, 30]. Therefore, the stratification in White and Black can reflect wrong genetic findings since ancestry is an important factor in ALS [27, 31].

For lifestyle has also been investigated by several studies as possible risk factors for the disease [5, 32, 33]. Researches indicate that the smoking provides an increased risk factor for the development of ALS, due to the components of the cigarette that are toxic and may promote neuronal toxicity, inflammation, and oxidative stress [34–36]. However, our findings did not show any significant difference between ALS patients and the control group for smoking (p = 0.372).

Besides, alcohol intake has not shown a significant association with ALS development (p = 0.319). The relationship between alcohol consumption and ALS is not yet fully established and is controversial [32]. Some studies indicate alcohol as a protective factor to ALS [37, 38], while others suggest that chronic alcohol consumption promotes glutamate-induced excitotoxicity, oxidative stress and induces changes in the intestinal microbiota, promoting the release of bacteria into the bloodstream and consequent production of inflammatory (TNF-α, IL-1β, IL-6), and oxidative mediators, which can break the blood-brain barrier and cause brain damage [39, 40].

Evidences suggest that oxidative stress is a major contributor to the emergence and progression of neurodegenerative diseases through tissue damage, neurodegeneration, neuronal dysfunction, apoptosis, microglia, and astrocyte activation [41, 42]. In both sporadic ALS and familial ALS, impairment of the redox state is a crucial factor for the development and progression of death of motor neurons [42].

Dysfunctions in genes responsible for producing enzymes related to the detoxification system, such as *GSTs*, may contribute significantly to the ALS pathogenesis due to changes in the redox state. *GSTs* polymorphisms have already been described as risk factors for the pathogenesis of Alzheimer, Multiple Sclerosis and Parkinson [13, 15, 18]. Therefore, studies of *GST* polymorphism, such as *GSTP1* rs1695 in ALS may be crucial for elucidating the molecular and pathogenic mechanisms involved in the onset and progression of the disease, as well as assisting to develop effective therapeutic strategies.

GSTP1 is characterized as a single subclass of GSTs involved with cellular protection against oxidative stress elements in the central nervous system, acting in the inhibition of oxidative damage to proteins, lipids, and nucleic acids [15, 43]. Reduced levels of GSTP1 messenger RNA (mRNA) have been already observed in mice transgenic for SODG93A, an animal model of ALS [43]. In analysis of protein and mRNA expression of peripheral blood mononuclear cells from ALS patients had been showed reduced expression of GSTP1 [44].

Despite these descriptions, there is only one study describing the role of the *GSTP1* rs1695 polymorphism in ALS, two studies with patients with Motor Neuron Disease (MND), and there are no published data for a study conducted in Brazil [12, 45, 46]. This highlights the importance of studying the *GSTP1* rs1695 polymorphism in the Brazilian population, since different populations and ethnicities may have a genetic variation, and therefore different actions of the polymorphic variant, which may result in morphological and disease-related phenotypic diversity [47].

Genotypic frequencies of this study revealed that the case group had a lower predominance of A/G (41.6% versus 46.5% in controls) and G/G genotypes (5.9% versus 11.9% in controls). Concordantly, these observations was found in a study in patients with MND from Russia, where 48.3% had the genotype A/G (versus 47.62% in controls) and 10% were carriers of the genotype G/G (versus 6.6% in controls) [45].

For the allele frequencies analysis, a lower frequency of the G allele was found in patients with ALS (27% versus 35% in controls). The same was observed in a cohort of Polish patients with MND, where 31.3% and 33.0% of the case and control group had the G allele, respectively [46]. An American study demonstred that the polymorphic variants may alter the clearance rate of lead-induced oxidative stressors and thereby influence a lead-ALS association [12].

Furthermore, when evaluated the observed and expected genotypic frequencies of the case, control group and the total study population, the results showed that were within the HWE, a fundamental parameter for studies of genetic association (p>0.05). These findings support that the mating is random, there are no genotyping errors and there is no mutation, migration or population selection, allowing us to trust in findings [48].

For genetic inheritance models there was no risk association between the *GSTP1* rs1695 polymorphism and ALS (Table 4). These findings may be explained by the multifactorial ALS character that may result in the interactions of genes, aging, environmental exposure, lifestyle, and comorbid situations [9]. The non-association of *GSTP1* rs1695 polymorphism and ALS risk was also demonstrated in patients from New England [12]. Besides, in a study conducted in Russia and Polish it was demonstrated that *GSTP1* rs1695 genotypes were not involved in MND development [45, 46]. However, in these studies, analysis by genetic inheritance models was not used.

Another differential of our study was the comparison of the *GSTP1* rs1695 polymorphism genotypes with demographic and clinical characteristics, and risk factors for the disease. Regarding age, our findings demonstrated that patients with different genotypes had a similar average. However, we observed that the A/G and G/G genotypes were not related to an early age of the disease (Table 5). Agúndez and collaborators [16] also showed that the age of onset of Multiple Sclerosis was not statistically different between patients with rs1695 variant, which in turn is similar to our findings.

On the other hand, our findings demonstrated that G/G genotype and G allele are not a risk factors for death in ALS Brazilian patients (Table 6). Based in the literature, we may hypotezited that a variety of conditions could that influence the outcome, such older age, psychosocial factors, frontotemporal dementia, nutritional status, and respiratory function [49]. Furthermore, although genome-wide association studies have contributed directly to a better understanding of genetic diseases, GST polymorphisms were not yet identified as possible susceptibility genes for ALS [50].

In general, oxidative stress in ALS may result from environmental exposure [9, 11]. Oxidative stress is a crucial factor in the degeneration of motor neurons and oxidative damage to proteins, lipids, and DNA. These effects have been observed in postmortem patients with sporadic ALS in neuronal tissue analysis [51]. Several exogenous variables, such as smoking, alcohol intake, and exposure to chemical agents and heavy metals (lead, selenium, and mercury) have already been considered as a risk factor for ALS due to oxidative stress that induces the degeneration of motor neurons [9, 11, 12].

When we investigated the association for *GSTP1* rs1695 genotypes with environmental exposure, the results demonstrated that A/A genotype presented a significantly difference (p = 0.03) (Table 7). Longo et al. [15] described that exposure to pesticides was associated to Parkinson’s disease, being this effect increased in patients with heterozygote genotype for rs1695.

Lastly, Kaplan-Meier curves for *GSTP1* rs1695 genotypes are shown in Fig 2. For these analysis no significant difference was found, indicating that the G allele did not influence the cumulative survival of ALS patients in the Brazilian population. Studies involving rs1695 and neurological diseases are scarce, making population comparisons difficult. On the other hand, we encourage studies with these variables in different populations of ALS patients.

Considering all these findings, our data support that *GSTP1* rs1695 polymorphism is not associated with ALS risk in patients from the Brazilian central population. Further genetic association studies are needed to understand the relationship of polymorphisms related to the metabolization process and the pathophysiology of ALS. We hope that our results will be offering a basis for more understanding of the *GSTs* polymorphisms for ALS risk.

## Supporting information

S1 Fig Original gel of the genotyping of the *GSTP1* rs1695 polymorphism by PCR-RFLP. Lanes 1, 2, 9 and 10: Empty wells. Lane 3: Marker—Molecular DNA size marker 50bp (Sinapse 50bp DNA ladder). Lane 4: Wild genotype (A/A). Lane 5 and 6: Mutant genotype (G/G). Lane 7: Heterozygous genotype (A/G). Lane 8: Positive control (sample previously known as heterozygous genotype—A/G).
(TIF)
