# Supplementary figures and images for: No association of GSTP1 rs1695 polymorphism with amyotrophic lateral sclerosis: A case-control study in the Brazilian population
Source: PLoS One. 2021 Feb 19;16(2):e0247024. doi: 10.1371/journal.pone.0247024 (PMC7894827; doi:10.1371/journal.pone.0247024)

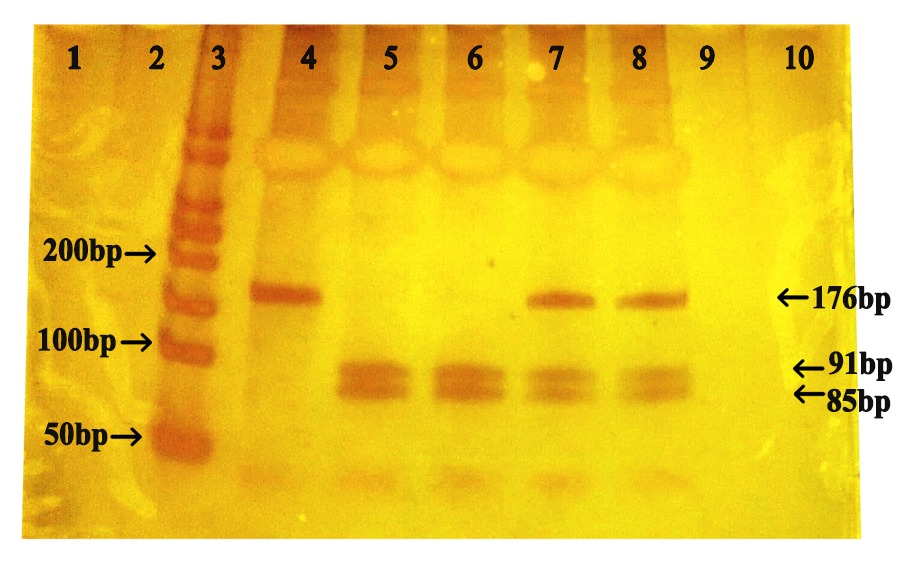

Supplement: S1 Fig — Lanes 1, 2, 9 and 10: Empty wells. Lane 3: Marker—Molecular DNA size marker 50bp (Sinapse 50bp DNA ladder). Lane 4: Wild genotype (A/A). Lane 5 and 6: Mutant genotype (G/G). Lane 7: Heterozygous genotype (A/G). Lane 8: Positive control (sample previously known as heterozygous genotype—A/G). (TIF) [file pone.0247024.s001.tif]
